# Predictors of subjective well-being in Korean men and women: Analysis of nationwide panel survey data

**DOI:** 10.1371/journal.pone.0263170

**Published:** 2022-02-10

**Authors:** Inmyung Song, Hye-Jae Lee

**Affiliations:** 1 College of Nursing and Health, Kongju National University, Gongju, Korea; 2 College of Pharmacy, Woosuk University, Wanju, Korea; University of Salamanca, SPAIN

## Abstract

Subjective well-being has been associated with sociodemographic characteristics, health, and satisfaction with family life. There is evidence on gender difference in subjective well-being and differential relationships of predictors between men and women worldwide. However, little is known about the gender gaps in subjective well-being in Korean adults. Using nationwide panel survey data, this study aims to examine predictors of subjective well-being in the Korean population and to investigate if there is a difference in the impact of some predictors between men and women. Generalized estimating equations were used to measure the relationship between subjective well-being and explanatory variables, using individual-level data from the Korean Welfare Panel Study (KOWEPS) between 2017 and 2020. Model 1 investigated sociodemographic variables. Model 2 added three health-related variables (such as disability, chronic disease, and subjective health status) and satisfaction with family life. Additional models included a range of interaction terms. In the 2020 KOWEPS, 10,758 respondents rated their subjective well-being scores on the Cantril ladder. The mean score of all respondents was 6.74 (SD = 1.66). In the analysis of the pooled sample, subjective well-being was higher in women than in men (Models 1–2, *p* < .01). Among all variables examined, satisfaction with family life was the most important predictor of subjective well-being (β = 1.3625; *p* < .01). Education level and employment status had significant interaction effects with gender on subjective well-being. In particular, higher education was more important for women and stable employment was more important for men.

## Introduction

South Koreans, on average, appear to be less happy than residents in other industrialized nations [1, 2]. Such unhappiness may have detrimental consequences in terms of sick days, low productivity, and a higher risk of depression [3–5]. Furthermore, unhappiness and life dissatisfaction may be associated with high suicide rates that last for a long time [6, 7]. Whether the cause is collective unhappiness or not, South Korea recorded the world’s highest suicide rate of 23 per 100,000 population in 2017 [8]. In this context, it is imperative to examine which factors influence subjective well-being among Koreans.

There is a plethora of studies that attempt to identify factors influencing subjective well-being. First, subjective well-being is associated with sociodemographic factors such as sex, age, marital status, and education level [9–11]. There is a difference in subjective well-being of residents in urban and rural areas, although the direction of the relationship varies across countries [12, 13]. Financial circumstances such as household income [2, 11] and employment status [14] are shown to impact subjective well-being.

In addition to sociodemographic variables, health status is a key factor that influences subjective well-being [15]. Health status can be measured by disability status, chronic disease status, and subjective health status. First, disability status and the severity of impairment were negatively associated with subjective well-being among older married adults with disability in the United States [16]. In addition, an analysis of German and British panel study data showed that disability was associated with moderate to large drops in happiness, which did not recover to the baseline levels in the follow-up period of 3 to 7 years [17]. A study based on data of 21 European countries reported that the disability level did not explain inequality in subjective well-being [18]. Second, chronic disease was also negatively associated with subjective well-being. For example, U.K. women who were treated for chronic condition such as arthritis and depression were less likely to feel happy than those without chronic condition [19], and an analysis of Indonesian family survey data showed that the duration of chronic illness had a negative effect on happiness and evaluation of one’s life [20]. Third, self-reported health was one of the most important correlates of subjective well-being in many countries [21, 22]. Likewise, happiness was significantly associated with subjective rather than objective assessment of health [23].

Another key predictor of subjective well-being appears to be the quality of social interactions and family relationship [24, 25]. According to a study in Europe, satisfaction with family life appears more important than satisfaction with another domain of life, such as job, in explaining one’s happiness [26]. In recent decades, family structure and traditional family relations in South Korea have changed dramatically [27]. Family size has become smaller and an increasingly liberal atmosphere has contributed to greater qualities of family life. Nevertheless, satisfaction with family life was shown to be an important correlate with suicide ideation among Korean adults [28], suggesting the significance of examining the impact of the variable on subjective well-being. In this context, we sought to examine the role of satisfaction with family life on subjective well-being.

Among all these predictors, gender is the one that received wide attention with a particular focus on gender gap in subjective well-being [29, 30]. On average, women have higher subjective well-being than men around the world [31]. However, women’s well-being advantage does not appear to be universal in that the gender gap in subjective well-being was greater in wealthy countries and in poor countries [31]. Furthermore, men were happier than women in some countries [32].

The literature links gender differences in subjective well-being to gender inequality in social conditions such as education, employment status, and political freedom [33, 34]. However, the gender gap was not fully explained by observable characteristics of individuals, suggesting men and women perceive life conditions differently [35]. Moreover, some characteristics of individuals appear to have differential relationships with well-being between men and women. For example, the relationship between age and well-being differed between men and women [36]. More specifically, women start out happier than men in early adulthood but the reverse is true later in life. In addition, employment status and education level were stronger predictors of life satisfaction in men, whereas marital status and social support were stronger predictors in women [37]. Of social support, family relations appear to be more important for men, whereas social relationships with friends are more important for women [38, 39].

Despite this existing body of knowledge on subjective well-being around the world, there remains a gap in the understanding of factors that influence the subjective well-being of people in Korea. So far, gender difference research has been limited to certain age groups in the Korean population [40, 41]. It is therefore worth investigating what influences subjective well-being in Korean men and women, because the same factors influence subjective well-being differently depending on the context and have dissimilar relationships to subjective well-being between men and women across cultures [42]. This study therefore seeks to examine predictors of subjective well-being in the Korean population and to investigate if there is a difference in the impact of some predictors for men and women, using nationally representative panel survey data.

Having reviewed the literature, we formulated the following hypotheses. First, women have higher subjective well-being than men in Korea. Second, subjective well-being is associated with health and family satisfaction variables, after controlling for sociodemographic variables. Third, the relationship between key variables, such as education and employment status, and subjective well-being differs between men and women.

These hypotheses can be written as the following equations.

$${SWB}_{it}=\alpha +\sum_{j=1}^{8}\beta_{j}S_{it}+\sum_{j=9}^{12}\beta_{j}H_{it}+\mu_{i}+\epsilon_{it},$$ (1)

where SWB is subjective well-being score as a continuous variable; S_it_ is sociodemographic variables comprising sex, age, marital status, education, area of residence, low income family status, income, and employment type; H_it_ is health and satisfaction variables comprising disability, chronic disease, subjective health, and satisfaction with family life; μ_i_ and e_it_ are error terms.

$${SWB}_{it}=\alpha +\sum_{j=1}^{8}\beta_{j}S_{it}+\sum_{j=9}^{12}\beta_{j}H_{it}+{Sex}_{it}∙\sum_{k=1}^{7}\gamma_{k}S_{it}+{Sex}_{it}∙\sum_{k=8}^{11}\gamma_{k}H_{it}+\mu_{i}+\epsilon_{it},$$ (2)

where interaction terms between sex and sociodemographic, health, and satisfaction variables were added to Eq (1).

## Materials and methods

### Data source

To test for the hypotheses set forth, this study used individual-level data from the 12th to 15th waves of the Korean Welfare Panel Study (KOWEPS) in 2017–2020 for which subjective well-being was collected. The KOWEPS is a longitudinal study of a nationally representative sample of families conducted by the Korea Institute for Health and Social Affairs since 2005. The KOWEPS uses a stratified two-stage sampling method to select the panel families. In the first phase, 517 administrative districts were sampled from the South Korean census data and the household income data of residents were collected. In the second phase, families were stratified into general households and low-income households, which were defined as those whose income falls below 60% of the median household income. In 2020, the panel comprised 6,460 families, of which 6,029 completed the survey (response rate = 93.3%) [43]. The size of the panel who completed the survey has become smaller due to attrition from 6,581 in 2017, 6,474 in 2018, 6,331 in 2019, to 6,029 in 2020. From these families, the KOWEPS annually collects, through face-to-face interviews, a variety of data including demographic, perceived health status, life satisfaction, and subjective well-being.

### Subjective well-being

Subjective well-being encompasses a range of evaluations of one’s life, such as affective reactions to life events, job satisfaction, and life satisfaction [44]. Among these, life satisfaction represents how one appraises one’s life taken as a whole. Since 2017, the KOWEPS’s questionnaire, administered to individuals aged 15 years and older, included a new question to measure subjective well-being on the Cantril ladder scale [43]. Respondents were asked to imagine a ladder with rungs numbered from zero at the bottom to 10 at the top, where the top of the ladder represents the best possible life and the bottom the worst possible life. Respondents were then asked to indicate on which rung of the ladder they would feel they stand on the date of the survey. In 2020, a total of 10,758 respondents rated their subjective well-being on the Cantril ladder scale. The Cantril ladder scale has been treated as a reliable, theoretically equal interval and continuous measure of subjective well-being [45].

### Explanatory variables

Explanatory variables were selected based on a thorough review of the literature and drawing on the availability of variables in the KOWEPS dataset. First, this study examined a set of sociodemographic characteristics, such as sex, age, marital status, education level, area of residence, income status, annual household income, and employment type. In addition, the KOWEPS database provides information on disability status, duration of treatment for chronic disease, and subjective health status, all of which were utilized as measures of health status in the present study. Disability status represents whether the respondents experience any difficulty in performing daily activities. Chronic disease was defined as the condition that has been treated for at least 3 months, based on the National Cancer Institute’s definition [46]. In the KOWEPS, subjective health status was rated on a 5-point scale: 1 (very healthy), 2 (healthy), 3 (neutral), 4 (unhealthy), and 5 (very unhealthy). In this current study, subjective health status was re-categorized into a binary variable: healthy and not healthy. 1 and 2 were recategorized as healthy and all others as not healthy.

In the KOWEPS, respondents were further asked to rate how satisfied they were with their family lives on the date of the survey on a 7-point scale that ranged from 1 (very dissatisfied) 2 (dissatisfied), 3 (a little dissatisfied), 4 (neutral), 5 (a little satisfied), 6 (satisfied), to 7 (very satisfied). 5, 6, and 7 were re-categorized as satisfied and all others as not satisfied.

The relationship between age and subjective well-being was found to be quadratic [47]. Therefore, age squared was entered into the regression model. Given the literature pointing to the changing relationship between age and gender happiness gap over the course of life cycle [36], we added the interaction term of age*gender in the regression model.

### Statistical analyses

Mean subjective well-being scores of respondents were calculated according to sociodemographic characteristics, such as sex, age group, marital status, education level, area of residence, low income household status, and employment type. These univariate analyses were conducted using data for the most recent year (2020). Age was grouped into 15–24, 25–44, 45–64, and ≥ 65 years to represent life cycles of young adults, adults, middle aged, and aged [48]. Marital status was categorized into three groups: ever married, never married, and currently married. The divorced, the separated, and the widowed were all combined into the ever married group, as the number of respondents in the category is relatively small. Education level was categorized into three groups: primary or less, middle and high school, and college and more. Area of residence was categorized as Seoul metropolitan, other metropolitan, city, and rural. Employment type was categorized as salaried worker-permanent, salaried worker-temporary (i.e., contract workers), self-employed, and non-employed. The non-employed category comprised economically inactive individuals and the unemployed.

Mean subjective well-being scores were also calculated by disability status, chronic disease status, and subjective health status. The t-test and the analysis of variance (ANOVA) test were used to test differences in mean subjective well-being scores between different categories. Post-hoc pairwise comparisons were made by using the Tukey’s test.

To measure the relationship between subjective well-being and explanatory variables, we used a generalized estimating equation (GEE) approach as panel data may not satisfy the assumption that data are independent [49]. GEE is also regarded as a robust method to estimate more efficient and unbiased regression coefficients than ordinary least squares for repeated measures in panel dataset [50]. Having observed that scores were approximately normally distributed, we treated subjective well-being measured on the Cantril ladder as a continuous variable, as was done by other researchers [45]. In the GEE analysis, each year’s well-being score for an individual was treated as one observation. We used quasi-likelihood under the independence model criterion (QIC) as a model-selection method, which was viewed as an appropriate method for GEE [51].

A set of variables were added as predictors of subjective well-being in a succession of models. The initial model (Model 1) included only sociodemographic variables. Model 2 added health-related variables and satisfaction with family life. Regression analyses were performed separately for men and women, as well as for the pooled sample. In the pooled analysis, sex was considered by using a dummy variable (0 = male, 1 = female). In all regression models, age and annual household income (in $10,000, 1,150 KRW/USD) were entered as continuous variables. Dummy variables were created for all categorical variables. Interaction terms were created and added into a series of subsequent regression models.

Unequal selection probability is an issue inherent in complex survey data, which occurs due to oversampling of some subgroups of the population and poststratification adjustments for nonresponse [52]. Therefore, all the analyses are population weighted by applying sampling weights, which are the inverse of selection probability. Normalized weights (i.e., weights divided by the mean weight in the sample) were used for regression analyses. Regression coefficients and *p*-values were presented to show the magnitude and significance of the relationship between subjective well-being and predictor variables. SAS version 9.4 (Cary, NC, USA) was used for data processing and STATA 14 (College Station, TX, USA) was used for econometric model analysis. The study protocol was approved by the Institutional Review Board of Kongju National University (IRB no. KNU_IRB_2020–49). The need for consent was waived by the ethics committee.

## Results

In the weighted estimates for the 2020 KOWEPS, 51.38% of respondents were female and 39.82% were 45–64 years old (Table 1). The mean subjective well-being score of all respondents was 6.74 (SD = 1.66). There was no significant difference in subjective well-being between men and women. The mean subjective well-being score was lowest in respondents aged 65 years and older (M = 6.19, *p* < .001), and highest in currently married people (M = 6.97, *p* < .001), and in those with college or postgraduate education (M = 7.06, *p* < .001). The mean subjective well-being score was lowest in rural areas among all area groups and highest in permanent salaried workers among all employment types (*p* < .001).

**Table 1:** Mean subjective well-being scores according to sociodemographic characteristics in 2020 (n = 10,758).

Variable	Category	Respondents		Subjective well-being		p-value	99% CI
		N	%	Mean	SD		
Total		40,849,975	100.00	6.74	1.66		
Sex	Male	19,860,353	48.62	6.77	1.77	0.156	-0.04, 0.13
	Female	20,989,622	51.38	6.72	1.57		
Age group	15-24	2,883,115	7.06	6.94	1.66	<0.001	NA
	25-44	13,979,493	34.22	6.94	1.97		-0.18, 0.32
	45-64	16,266,987	39.82	6.80	1.84		-0.47, 0.01
	65+	7,720,379	18.90	6.19	1.20		-1.16, -0.68*
Marital status	Currently married	25,004,790	61.21	6.97	1.59	<0.001	NA
	Ever married	5,103,144	12.49	5.86	1.33		-1.11, -0.87*
	Never married	10,742,041	26.30	6.65	2.03		-0.28, -0.02*
Education	Primary education or less	4,782,339	11.71	5.78	1.16	<0.001	NA
	Middle and high school	15,002,077	36.72	6.61	1.61		0.67, 0.98*
	College or more	21,065,558	51.57	7.06	1.89		1.13, 1.43*
Area of residence	Seoul metro	7,906,084	19.35	6.46	1.86	<0.001	NA
	Other metro	9,625,249	23.56	6.51	1.71		-0.12, 0.22
	City	19,855,676	48.61	6.53	1.73		-0.09, 0.23
	Rural	3,462,967	8.48	6.27	1.71		-0.37, 0.00
Low income family	No	34,205,154	83.73	6.97	1.70	<0.001	NA
	Yes	6,644,821	16.27	5.58	1.30		1.29, 1.48*
Employment type	Salaried-permanent	12,754,062	31.22	7.23	1.38	<0.001	NA
	Salaried-temporary	9,056,253	22.17	6.47	1.66		-0.92, -0.61*
	Self-employed	5,180,333	12.68	6.55	1.57		-0.85, -0.52*
	Non-employed	13,859,326	33.93	6.02	1.87		-1.35, -1.09*

Abbreviations: CI, Confidence interval. NA, not available (reference).

The number of respondents in the sample was 10,758 but the values in the table were population estimates obtained by using sampling weights.

*p*-values were obtained from the t-test and the ANOVA test.

Ever married means divorced, separated, and widowed.

Low income family is the household whose equalized family income is under 60% of median in Korea. The non-employed category comprised economically inactive individuals as well as the unemployed.

*Post-hoc analysis was significant at the 0.01 level.

Of all respondents, 6.50% (n = 2,650,523) reported having a disability (Table 2). Those with disability, on average, reported a lower subjective well-being score than those without disability (M = 5.75 for respondents with disability and M = 6.81 for those without disability, *p* < .001). Respondents with chronic disease (M = 6.46) reported a lower subjective well-being score than those without chronic disease (M = 6.96, *p* < .001). The mean subjective well-being score was higher in respondents who rated they were healthy and those who were satisfied with family life (*p* < .001).

**Table 2:** Mean subjective well-being scores by health status and satisfaction with family life in 2020 (n = 10,758).

Variable	Category	Respondents		Subjective well-being		p-value	99% CI
		N	%	Mean	SD		
Total		40,849,975	100.00	6.74	1.66		
Disability	No	38,199,452	93.51	6.81	1.64	<0.001	0.90, 1.22*
	Yes	2,650,523	6.49	5.75	1.60		
Chronic disease	No	23,367,777	57.20	6.96	1.77	<0.001	0.42, 0.58*
	Yes	17,482,198	42.80	6.46	1.54		
Subjective health	Not healthy	11,620,474	28.45	6.06	1.49	<0.001	-1.05, -0.87*
	Healthy	29,229,500	71.55	7.02	1.68		
Satisfaction with family life	Not satisfied	7,736,378	18.94	5.26	1.70	<0.001	-1.82, -1.61*
	Satisfied	33,113,597	81.06	7.03	1.46		

Abbreviation: CI, Confidence interval. Values were adjusted with sampling weights.

The number of respondents in the sample was 10,758 but the values in the table were population estimates obtained by using sampling weights.

*p-*values were obtained from the t-test and the ANOVA test.

* Post-hoc analysis was significant at the 0.01 level.

In a multiple regression analysis of the pooled sample, when other variables were adjusted for, women were shown to have higher subjective well-being scores than men (Models 1–2, *p* < .01) (Table 3). While age had a negative impact (*p* < .01 in Model 1), it had a quadratic relationship with subjective well-being (*p* < .01 in Model 1 and *p* < .05 in Model 2). Ever married or never married individuals reported lower subjective well-being scores than currently married people (*p* < .01). Subjective well-being increased with more education (*p* < .01). Residents in the Seoul metropolitan area had lower subjective well-being than those in other metro and rural areas (*p* < .01). Subjective well-being was lower in low income families than in general households, and increased with income (*p* < .01). Permanent salaried workers reported higher subjective well-being scores than individuals with any other employment status (*p* < .01). Subjective well-being was associated negatively with disability (*p* < .01) but positively with subjective health status (*p* < .01) in Model 2. Satisfaction with family life had a stronger impact on subjective well-being than any other variable (β = 1.3625, *p* < .01).

**Table 3:** Factors associated with subjective well-being in men and women.

Variable (reference)		Model 1		Model 2	
		β	SE	β	SE
**Sociodemographic variables**					
Sex (male)	Female	0.1859***	0.0236	0.1380***	0.0209
Age	Age	-0.0332***	0.0048	-0.0028	0.0044
	Age^2^	0.0003***	0.0000	0.0001**	<0.0001
Marital status (currently married)	Ever married	-0.3396***	0.0327	-0.2308***	0.0287
	Never married	-0.6298***	0.0429	-0.3048***	0.0391
Education (≤ primary)	Middle and high school	0.2236***	0.0351	0.1639***	0.0304
	College and more	0.6478***	0.0446	0.4797***	0.0394
Area of residence (Seoul metro)	Other metro	0.1100***	0.0365	0.0733**	0.0317
	City	0.0875**	0.0347	0.0365	0.0301
	Rural	0.2365***	0.0396	0.1264***	0.0349
Low income family (no)	Yes	-0.3057***	0.0360	-0.2028***	0.0321
Income		0.4223***	0.0227	0.2911***	0.0205
Employment type (salaried-permanent)	Salaried-temporary	-0.3039***	0.0317	-0.2513***	0.0286
	Self-employed	-0.1704***	0.0340	-0.1498***	0.0305
	Non-employed	-0.4588***	0.0315	-0.3231***	0.0281
**Health and satisfaction variables**					
Disability (no)	Yes			-0.1400***	0.0350
Chronic disease (no)	Yes			0.0147	0.0223
Subjective health (not healthy)	Healthy			0.4809***	0.0212
Satisfaction with family life (not satisfied)	Satisfied			1.3625***	0.0218
Constant		6.6634***	0.1565	4.5507***	0.1441
QIC		113,642.28		98,396.48	

Note:

* *p*<0.1

** *p*<0.05

*** *p*<0.01.

Income (USD 10,000) was log-transformed.

The results of subgroup analyses by gender showed that among sociodemographic variables, college and post-graduate education had a stronger influence on well-being for women than for men (β = 0.5623 and β = 0.3712, respectively, in Model 2) (Tables 4 and 5). On the other hand, employment type had a stronger influence on well-being for men than for women. In particular, the β coefficient for non-employment was -0.5234 for men and -0.2155 for women in Model 2 (*p* < .01). Among health variables, subjective health status had a significant impact on well-being in both men and women, but disability was negatively associated with well-being only in women (*p* < .01).

**Table 4:** Factors associated with subjective well-being in men.

Variable (reference)		Model 1		Model 2	
		β	SE	β	SE
**Sociodemographic variables**					
Age	Age	-0.0708***	0.0072	-0.0362***	0.0065
	Age^2^	0.0006***	0.0001	0.0004***	0.0001
Marital status (currently married)	Ever married	-0.5052***	0.0573	-0.2403***	0.0514
	Never married	-0.6328***	0.0579	-0.3205***	0.0533
Education (≤ primary)	Middle and high school	0.1694***	0.0534	0.0872*	0.0469
	College or more	0.5280***	0.0645	0.3712***	0.0568
Area of residence (Seoul metro)	Other metro	0.1645***	0.0546	0.1100**	0.0478
	City	0.1661***	0.0526	0.1016**	0.0461
	Rural	0.2561***	0.0601	0.1623***	0.0534
Low income family (no)	Yes	-0.2296***	0.0538	-0.1408***	0.0484
Income		0.4694***	0.0346	0.3535***	0.0318
Employment type (salaried-permanent)	Salaried-temporary	-0.3827***	0.0472	-0.3137***	0.0428
	Self-employed	-0.2605***	0.0457	-0.2229***	0.0412
	Non-employed	-0.6983***	0.0511	-0.5234***	0.0462
**Health and satisfaction variables**					
Disability (no)	Yes			-0.0852*	0.0506
Chronic disease (no)	Yes			0.0432	0.0318
Subjective health (not healthy)	Healthy			0.4333***	0.0313
Satisfaction with family life (not satisfied)	Satisfied			1.2815***	0.0335
Constant		7.6663***	0.2241	5.5028***	0.2098
QIC		115,893.79		99,553.43	

Note:

* *p*<0.1

** *p*<0.05

*** *p*<0.01.

Income (USD 10,000) was log-transformed.

**Table 5:** Factors associated with subjective well-being in women.

Variable (reference)		Model 1		Model 2	
		β	SE	β	SE
**Sociodemographic variables**					
Age	Age	-0.0067	0.0066	0.0206***	0.0059
	Age^2^	0.0001	0.0001	-0.0001**	<0.0001
Marital status (currently married)	Ever married	-0.2424***	0.0414	-0.1975***	0.0361
	Never married	-0.5522***	0.0644	-0.2198***	0.0582
Education (≤ primary)	Middle and high school	0.2727***	0.0476	0.2032***	0.0411
	College or more	0.7670***	0.0627	0.5623***	0.0555
Area of residence (Seoul metro)	Other metro	0.0691	0.0487	0.0496	0.0422
	City	0.0347	0.0459	-0.0054	0.0396
	Rural	0.2228***	0.0524	0.1024**	0.0458
Low income family (no)	Yes	-0.3547***	0.0479	-0.2452***	0.0426
Income		0.3924***	0.0299	0.2500***	0.0266
Employment type (salaried-permanent)	Salaried-temporary	-0.2105***	0.0437	-0.2014***	0.0394
	Self-employed	-0.0643	0.0519	-0.0888*	0.0463
	Non-employed	-0.2935***	0.0420	-0.2155***	0.0375
**Health and satisfaction variables**					
Disability (no)	Yes			-0.1755***	0.0483
Chronic disease (no)	Yes			-0.0126	0.0314
Subjective health (not healthy)	Healthy			0.5178***	0.0286
Satisfaction with family life (not satisfied)	Satisfied			1.4045***	0.0286
Constant		6.0391***	0.2148	3.9775***	0.1963
QIC		114,501.60		98,951.10	

Note:

* *p*<0.1

** *p*<0.05

*** *p*<0.01.

Income (USD 10,000) was log-transformed.

Table 6 presents three models examining interaction effects. Interaction terms between sex and sociodemographic variables were in Model 1. Interaction terms between sex and health and satisfaction variables were in Model 2. Model 3 comprises all interaction terms. Gender and education level had a significant interaction effect on subjective well-being (*p*<0.01 in Models 1 and *p*<0.05 for college education in Model 3). There was also a positive interaction effect between non-employment and female (β = 0.2254, *p*<0.01 in Model 3).

**Table 6:** Factors associated with subjective well-being including interaction effects.

Variable (reference)		Model 1		Model 2		Model 3	
		β	SE	β	SE	β	SE
**Sociodemographic variables**							
Sex (male)	Female	0.1456	0.1748	0.0530	0.0558	-0.0186	0.1839
Age		0.0075***	0.0015	0.0064***	0.0010	0.0067***	0.0015
Marital status (currently married)	Ever married	-0.2293***	0.0513	-0.2194***	0.0288	-0.2399***	0.0517
	Never married	-0.1817***	0.0498	-0.2659***	0.0344	-0.1917***	0.0501
Education (≤ primary)	Middle and high school	0.0353	0.0457	0.1456***	0.0303	0.0404	0.0461
	College or more	0.3485***	0.0563	0.4727***	0.0396	0.3614***	0.0569
Area of residence (Seoul metro)	Other metro	0.1090**	0.0477	0.0739**	0.0317	0.1106**	0.0479
	City	0.1087**	0.0460	0.0392	0.0301	0.1110**	0.0462
	Rural	0.1766***	0.0534	0.1300***	0.0348	0.1802***	0.0535
Low income family (no)	Yes	-0.1110**	0.0484	-0.1993***	0.0321	-0.1174**	0.0485
Income		0.3368***	0.0314	0.2890***	0.0204	0.3438***	0.0315
Employment type (salaried-permanent)	Salaried-temporary	-0.3046***	0.0428	-0.2606***	0.0288	-0.3098***	0.0430
	Self-employed	-0.2154***	0.0412	-0.1581***	0.0306	-0.2187***	0.0413
	Unemployed	-0.4382***	0.0445	-0.3219***	0.0278	-0.4526***	0.0447
**Health and satisfaction variables**							
Disability (no)	Yes	-0.1458***	0.0349	-0.1248**	0.0502	-0.1222**	0.0505
Chronic disease (no)	Yes	0.0154	0.0224	0.0169	0.0305	0.0365	0.0319
Subjective health (not healthy)	Healthy	0.4780***	0.0212	0.4406***	0.0302	0.4257***	0.0314
Satisfaction with family life (not satisfied)	Satisfied	1.3666***	0.0218	1.3271***	0.0332	1.3154***	0.0336
**Interaction terms**							
Female × age		-0.0011	0.0020			0.0004	0.0021
Female × marital: ever married		0.0191	0.0623			0.0307	0.0628
Female × marital: never married		-0.1227*	0.0696			-0.1020	0.0698
Female × education: middle and high school		0.1905***	0.0610			0.1804***	0.0614
Female × education: college or more		0.2290***	0.0789			0.2026**	0.0795
Female × residence: other metro		-0.0594	0.0639			-0.0615	0.0639
Female × residence: city		-0.1169*	0.0608			-0.1208**	0.0608
Female × residence: rural		-0.0784	0.0704			-0.0862	0.0704
Female × row income family		-0.1433**	0.0645			-0.1310**	0.0646
Female × income		-0.0800*	0.0411			-0.0924**	0.0413
Female × employment: salaried-temporary		0.1041*	0.0583			0.1111*	0.0583
Female × employment: self-employed		0.1304**	0.0620			0.1361**	0.0621
Female × employment: non-employed		0.2053***	0.0580			0.2254***	0.0580
Female × disability				-0.0456	0.0691	-0.0480	0.0699
Female × chronic disease				-0.0096	0.0406	-0.0414	0.0447
Female × healthy				0.0694*	0.0397	0.0905**	0.0425
Female × satisfied				0.0694	0.0437	0.0850*	0.0442
Constant		4.2639***	0.1303	4.3665***	0.0979	4.3614***	0.1348
QIC		98,319.73		98,393.26		98,293.68	

Note:

* *p*<0.1

** *p*<0.05

*** *p*<0.01.

Income (USD 10,000) was log-transformed

## Discussion

Using nationally representative panel survey data, this study showed that women had greater subjective well-being levels than men when other covariates were adjusted for. This finding is consistent with observations in other countries [31]. In a model adjusting for life conditions such as income and education, women had greater life satisfaction than men in many developing countries [53]. Such gender differences in well-being suggest that women react to life circumstances differently than men, and that women are happier than men despite the life circumstances, such as income, education, and health, which were all examined in our regression models. Besides gender differences, this study also revealed that satisfaction with family life was the most important predictor of subjective well-being in Korea and that some predictor variables influenced subjective well-being differently between men and women.

The current study examined the relationship between a range of sociodemographic variable and subjective well-being, with results confirming or refuting previous findings depending on variables. For example, the literature reports inconsistent relationships between age and subjective well-being; U-shaped [9], inverted U-shaped, or even linear [54, 55]. The present study showed that there was a nonlinear, U-shaped relationship between age and subjective well-being in Korea, as indicated by the positive coefficient for age^2^ in Tables 3 and 4. It was also shown that the relationship between age and subjective well-being did not differ between men and women, unlike what was suggested in a previous study [56]. The present study showed that being married was positively associated with subjective well-being, consistent with the findings of previous studies [22, 57]. The literature suggests that the relationship between marital status and subjective well-being differ by gender [42, 58]. For example, marital status is a strong predictor of happiness for men but not for women in East Asian countries [42]. In the United States, being married had a stronger impact on happiness for women than for men [58]. However, in the present study, the impact of marital status on subjective well-being did not differ between men and women, as indicated by insignificant interaction terms.

Educational attainment can increase happiness directly or indirectly by improving the chance of employment and a higher income [59], and by means of greater health status and healthier lifestyles [60]. The present study confirmed the positive relationship between education level and subjective well-being, which differed by gender. More specifically, the positive impact of higher education was greater for women than for men. The literature points to several possibilities for the difference in the effect of education on well-being [61, 62]. For example, women gain more health benefits from education than men [61]. In addition, education has a greater impact on self-esteem for women than for men [62]. Regardless of what led to gender differences, these findings suggest that there is a potential for well-being gain by promoting education in women, especially older women in Korea who might have experienced inequality in access to education in the past decades [63]. Future research could examine gender differences further in regards to what mediates the impact of education on subjective well-being.

There is evidence on differences in subjective well-being between urban and rural areas [12, 13]. In the present study, the results of multiple regression analyses showed that subjective well-being was lowest among residents in the Seoul metropolitan area but highest among those in rural areas. Similar observations were previously made among middle-aged and older Koreans [64]. The reasons for the findings are not known but only to be speculated. It is possible that lower subjective well-being in Seoul metropolitan areas may be attributable to a number of factors, such as long commutes [65], unaffordable housing prices [66], and lack of support from kin [67].

Subjective well-being depends on relative income as well as absolute income [2]. In the present study, subjective well-being was independently associated with two economic indicators reflective of relative and absolute income; subjective well-being was lower in low income households and increased with household income. These two economic indicators have a similar relationship with subjective well-being in both genders. On the contrary, the relationship between employment type and well-being differed between men and women. For example, non-employment had a negative main effect on subjective well-being but a positive interaction effect with female dummy variable. This suggests that while stable employment was positively associated with subjective well-being, consistent with the findings of previous studies [14, 68], non-employment does not influence negatively women’s well-being as much as it does men’s. The gender difference may arise from a greater detrimental effect that unemployment or underemployment may have on social approval in men than in women [69].

In addition to sociodemographic predictors, both physical and mental health were associated with happiness in many countries [7, 70]. However, having a serious illness or being treated for chronic conditions were not as important as self-rated general health in subjective well-being [19]. Similarly, the present study showed that among all health variables, subjective health status had the strongest influence on well-being. On the other hand, chronic disease status did not have any influence on well-being and disability had a negative influence on well-being in only some of the models examined. This may be a result of psychosocial adaptation to chronic condition, which could lower overall psychological distress and improve overall life satisfaction [71].

Even more important than perceived health status for subjective well-being was self-assessed close relationships with family and friends, according to a comprehensive 2012 survey in six countries [72]. The quality of family relations, such as spending more time with one’s family, may influence subjective well-being either directly or by means of a positive impact on physical health [73]. Among all variables examined in the present study, satisfaction with family life was the strongest predictor of subjective well-being in both men and women. The predictor appears to be more important for women than for men [42, 74], particularly, in countries where traditional gender ideology prevails [26]. However, in this present study, the relationship between subjective well-being and satisfaction with family did not vary between men and women.

So far research in the field of gender gap in subjective well-being in Korea has focused on the measure of life-satisfaction in the elderly with multimorbidity [41] and college students [40]. This present study enhances the current understanding of subjective well-being by gender in Korea, based on latest panel survey data using a large, nationally representative sample of the general population.

### Limitations

Despite the strengths of this study, this study has several limitations. First, the specification of the model was constrained by the availability of variables in the dataset and therefore could not examine the impact of other potential predictors of well-being, such as social capital and work-life balance. Some variables reflecting social capital derived from kin, friends and community were collected only in the first year (2005) of the KOWEPS and therefore could not be used in the present study, which analyzed data of four recent years in which subjective well-being scores were collected. Second, GEE provides only estimates of average response and does not warrant drawing causal inferences between predictors and subjective well-being. It would be of great interest for future research to examine the effect of transitions in some of the key variables, such as employment status and marital status, on subjective well-being over time to take advantage of the longitudinal nature of KOWEPS data. Third, we fitted GEEs to subjective well-being scores, having visually checked that values had a near normal distribution. However, we acknowledge that the scores are not by definition normally distributed, and that the assumption of normal distribution may have been too stringent. Although we used QIC for model selection, GEE does not have a universally accepted goodness-of-fit test, which is often regarded as an issue [50]. Future research could address the limitations by exploring different sources of data.

## Conclusion

In conclusion, this study showed that women had greater subjective well-being than men and that among all variables examined, satisfaction with family life was the most important predictor of subjective well-being in both men and women. In addition, the models including interaction terms showed that education level and employment type had differential impacts on subjective well-being between men and women. While it would be difficult to formulate specific implementable interventions to improve subjective well-being, our findings offer some important insights as to which areas to focus on to improve subjective well-being in the Korean population that is affected by the world’s highest suicide rates. One potential area that could have a considerable impact on well-being is improving satisfaction with family life, perhaps by building supportive environments for the family. Furthermore, efforts to improve subjective well-being should be sensitive to the needs of different groups. In particular, interventions to promote higher education would be more beneficial for women and policies to improve job security would provide greater benefits for men.
